# Review of current status and ongoing reforms of the mental health system in Uzbekistan

**DOI:** 10.1192/bji.2024.27

**Published:** 2025-02

**Authors:** Evgeniy Smekhov

**Affiliations:** Division of Public Health Science, Westminster International University in Tashkent, Tashkent, Republic of Uzbekistan, email: smekhov@mail.ru

**Keywords:** Mental health, Uzbekistan, regulatory, country profile, legislation, WHO guidelines

## Abstract

Uzbekistan's mental health system is undergoing significant reform aimed at improving care and service delivery. This article provides a comprehensive evaluation of the current state of Uzbekistan's mental health system using the WHO 2010 monitoring and evaluation framework. It also details ongoing reforms designed to address systemic issues, enhance mental healthcare and ensure better health outcomes for the population.

Uzbekistan, a central Asian nation with a population of approximately 37 million people,^1^ manages its health system through the Ministry of Health. This centralised system is predominantly public and places a strong emphasis on health services, including mental healthcare. Mental health services are primarily delivered through specialised psychiatric institutions, which are overseen by the Department of Mental Health within the Ministry of Health.^2^ The centralised model aims to streamline service delivery but also presents challenges in terms of resource allocation and service integration.

Uzbekistan's mental health system faces significant challenges including insufficient funding, stigma associated with mental illness and a shortage of trained mental health professionals. Despite the existence of a legislative framework that includes the Law on Psychiatric Care and a national suicide prevention strategy, these challenges hinder the effective delivery of mental health services.^3^ The existing infrastructure is often inadequate, and there is limited integration of mental health services into primary care. The primary objective of this article was to critically assess the current mental health system in Uzbekistan using the WHO 2010 framework.^4^ In addition, the article provides an overview of the ongoing reforms, evaluates their potential impact and offers some recommendations for the future.

## Method

We explored how effective the current mental health services in Uzbekistan are, as well as the impact of recent reforms on the quality and accessibility of mental healthcare. We followed the WHO 2010 framework, which evaluates health systems through six key building blocks: service delivery, health workforce, health information systems, access to essential medicines, financing and governance. Data were collected from various sources, including legislative documents, policy reports and interviews with key stakeholders in the mental health sector. The analysis involved evaluating the alignment of reforms with WHO guidelines, assessing the effectiveness of these reforms, and identifying gaps and areas for improvement.

## Results and discussion

Mental health services in Uzbekistan are managed by the Department of Mental Health within the Ministry of Health^3^ and include specialised psychiatric hospitals, out-patient clinics and community-based services.^3^ However, the system faces several challenges, including inadequate funding, limited access to services and insufficient infrastructure.

Accessibility to mental health services is constrained by financial limitations and geographical disparities. Psychiatric facilities are often concentrated in urban areas, leaving rural populations underserved. The readiness of services is affected by a shortage of resources and outdated infrastructure. Moreover, financial constraints have resulted in drug shortages and a reduction in the number of psychiatric beds.^5^ For instance, recent budget cuts have led to the closure of several psychiatric wards and a decrease in available medications. In addition, the integration of mental health services into primary healthcare remains underdeveloped, limiting the reach of mental health interventions. Recent reforms, including the establishment of a unified vertical management system and efforts to enhance integration with primary care, aim to address these issues. The reforms also focus on strengthening services for children and adolescents and improving infrastructure.^6^ The full impact of these reforms is yet to be determined, but initial steps include policy adjustments and infrastructural improvements.

Regarding the health workforce, Uzbekistan faces a significant shortage of trained mental health professionals: as of 2009, Uzbekistan had 937 psychiatrists and more than 2000 psychiatric nurses, resulting in a psychiatrist-to-nurse ratio of approximately 1:2. This is below the WHO-recommended ratio of 1:4. The workforce also lacks clinical psychologists and social workers, which are essential for comprehensive mental healthcare.^5^ The government's recent decree aims to address these shortages by enhancing training programmes and increasing incentives for mental health professionals. Since 1 September 2023,^6^ the government has funded training programmes and offered state grants for advanced education in psychiatry and related fields, alongside increasing financial incentives.

Strengthening health information systems is critical for effective mental healthcare. Recommendations include implementing electronic health records and developing robust monitoring and surveillance systems^7^ to track mental health conditions and patient outcomes. Data collection strategies guided by the WHO mhGAP toolkit will help with monitoring of service delivery and outcomes.

In terms of access to essential medicines, essential psychotropic medications are provided free of charge in mental hospitals, with at least 80% of the cost covered. The cost of antipsychotic medications represents 28% of the minimum daily wage, compared with 10% for antidepressants. These provisions are part of the disability benefits for people with mental disorders. Despite generally good access, challenges include ensuring consistent availability and addressing supply chain issues. The recent decree aims to address these challenges by ensuring full provision of necessary medications based on patient needs.

Uzbekistan's health expenditure was 5.9% of GDP in 2012, with 3% allocated to mental health services. Although this allocation is comparable with those of other Central Asian countries, it is lower than the European average.^8^ The recent decree mandates an increase in funding to 10 billion soums in 2023 for preferential medicines. From 2024, additional funds will be allocated based on state budget parameters. The funding strategy includes exploring cost management and reimbursement systems to ensure sustainable financing for mental health services.

Uzbekistan has established a comprehensive legislative framework for mental health, including key laws and presidential decrees, such as the Law on Psychiatric Care, Resolution of the Cabinet of Ministers No. 207, and Presidential Decrees Nos. 3606, PP-4190 and PP-196. Effective governance is essential for the successful implementation and sustainability of mental health reforms. Proposed indicators for monitoring governance include measures of service quality, implementation of reforms and adherence to legislative requirements.^9^

The ongoing reforms show promise in addressing systemic issues and improving mental healthcare. Strengths include increased funding, legislative support and a focus on integrating mental health services into primary care. However, challenges including financial constraints, implementation delays and resource limitations persist. Comparisons with other countries reveal areas for improvement, particularly in workforce training and service integration. Strategies for addressing these challenges include enhancing training programmes, strengthening health information systems and ensuring consistent funding.

## Conclusion

This article provides a detailed review of the current status and ongoing reforms of Uzbekistan's mental health system. Key challenges, including financial constraints and infrastructure limitations, are highlighted, alongside recent improvements such as increased funding and legislative support. Future efforts should focus on effectively implementing reforms, enhancing workforce training and strengthening data systems to improve mental healthcare.

## Data Availability

Data availability is not applicable to this article as no new data were created or analysed in this study.
